# Malícia honey (*Mimosa quadrivalvis* L.) produced by the jandaíra bee (*Melipona subnitida* D.) shows antioxidant activity via phenolic compound action in obese rats

**DOI:** 10.3389/fnut.2025.1524642

**Published:** 2025-02-06

**Authors:** Maria Luiza Rolim Bezerra, Mirela Gouveia-Nhanca, Maria Letícia da Veiga Dutra, Kamila Sabino Batista, Alana Natalícia Vasconcelos de Araújo, Marcos dos Santos Lima, Mateus Duarte Ribeiro, Alexandre Sergio Silva, Adriano Francisco Alves, Tatiana Colombo Pimentel, Marciane Magnani, Jailane de Souza Aquino

**Affiliations:** ^1^Experimental Nutrition Laboratory—LANEX, Department of Nutrition, Federal University of Paraíba (UFPB), João Pessoa, Brazil; ^2^Post Graduate Program in Nutrition Sciences, Federal University of Paraíba (UFPB), João Pessoa, Brazil; ^3^Semi Arid National Institute (INSA), Campina Grande, Brazil; ^4^Department of Food Technology, Instituto Federal do Sertão Pernambucano (IFSertãoPE), Petrolina, Brazil; ^5^Post Graduate Program in Food Sciences and Technology, Federal University of Paraíba (UFPB), João Pessoa, Brazil; ^6^Laboratory of Applied Studies in Physical Training to Performance and Health (LETFADS), Department of Physical Education, Federal University of Paraíba (UFPB), João Pessoa, Brazil; ^7^Associate Post Graduate Program in Physical Education (UPE/UFPB), Department of Physical Education, Federal University of Paraíba (UFPB), João Pessoa, Brazil; ^8^Laboratory of General Pathology, Department of Physiology and Pathology, Federal University of Paraíba (UFPB), João Pessoa, Brazil; ^9^Instituto Federal do Paraná (IFPR), Campus Paranavaí, Paranavaí, Brazil

**Keywords:** cafeteria diet, obesity, oxidative stress, phenolic compounds, stingless bee honey

## Abstract

**Background and aims:**

Obesity is a disease associated with increased oxidative stress in humans and animals, and consumption of antioxidant compounds such as polyphenols can minimise it. These compounds are abundant in malícia (*Mimosa quadrivalvis* L.) honey produced by stingless bees. This study aimed to evaluate whether administration of *Mimosa quadrivalvis* L. honey to obese rats could reduce oxidative stress in vital organs through phenolic compound action.

**Methods:**

Wistar rats (228 ± 14.69 g) were randomly divided into two groups: a healthy group (HG, *n* = 20) fed a control diet and an obese group (OG, *n* = 20) fed a cafeteria diet for the initial 8 weeks. After this period, these groups were again randomised into four subgroups: healthy (HG, *n* = 10), obese (OG, *n* = 10), healthy with malícia honey administration (1,000 mg/kg; HGH, *n* = 10), and obese with malícia honey administration (1,000 mg/kg; OGH, *n* = 10) for the final 8 weeks fed the previously mentioned diets. The rats were euthanised at the end of the experiment to collect brain, gut, kidney, and liver tissues to evaluate parameters related to oxidative stress and phenolic profile.

**Results:**

The administration of malícia honey reduced energy intake and weight gain in the OGH in comparison to the OG. Total antioxidant capacity increased in the brain, liver, and gut in both groups treated with honey compared to respective controls. Lipid peroxidation decreased in the brain, gut, and kidney of the OGH. Both treated groups showed elevated phenolic compound deposition, including catechin, procyanidins, and flavonoids, across all organs. Specifically, the brain in the OGH showed greater procyanidin B2 and gallic acid deposition; the liver showed increased procyanidin B1 and B2, epicatechin, and myricetin concentrations; the gut showed higher procyanidin B2 and kaempferol 3-glucoside concentrations; and the kidneys had increased catechin, procyanidin B1 and B2, and gallic acid deposition compared to the OG.

**Conclusion:**

Histologically, the OGH displayed reduced neuronal damage and prevention of hepatic steatosis induced by the cafeteria diet. Malícia honey effectively reduced oxidative stress via modulation of phenolic compounds in the brain, gut, kidney, and liver of cafeteria diet-induced obese rats.

## 1 Introduction

Obesity is a global health issue with high prevalence in high-income and middle-income countries. It is characterised by abnormal fat accumulation (1) and associated with elevated chronic inflammation and oxidative stress levels, which represents a risk for developing other non-communicable diseases, such as cardiovascular disease, metabolic syndrome, diabetes mellitus, hypertension, and hyperlipidaemia (2).

Previous studies have shown that the increased fat mass is positively associated with systemic oxidative stress in human and animal models (3). In turn, increased oxidative stress can stimulate fatty tissue deposition (4). Furthermore, hypercaloric cafeteria diet-induced obesity in young rats resulted in increases in free radical production, antioxidant enzyme activity, lipoperoxidation, catalase activity, and total glutathione levels in serum, which indicates oxidative stress (5).

In this scenario, consuming antioxidant compounds in a healthy diet could improve oxidative stress, such as phenolic compounds (6). There are three mechanisms by which phenolic compounds remove reactive oxygen species (ROS) which in turn reduces oxidative stress; first, through the transfer of hydrogen atoms; second, one electron transfer coupled with proton loss; and subsequent proton loss associated with an electron transfer (7).

Phenolic compounds are largely found in fruits and vegetables (6, 7); however, honey also can be considered a good dietary source of these compounds (8). Although honey produced by bees of the *Apis* species is widely consumed, commercialised, and studied, honey produced by stingless bees of the *Melipona* genus has gained prominence due to its nutritional and sensory characteristics and biological activity (9, 10). Honey produced by stingless bees has a variety of antioxidant compounds, including phenolic acids and flavonoids such as gallic acid, caffeic acid, chlorogenic acid, naringin, and taxifolin, and therefore it has therapeutic potential against oxidative stress (8). Nevertheless, the amount and profile of these compounds vary according to the bee species and local flora (8).

Among the honeys produced by stingless bees, monofloral malícia honey (*Mimosa quadrivalvis* L.) stands out. This honey is produced by jandaíra bees (*Melipona subnitida*), which are stingless endemic to the Brazilian Caatinga biome. These bees exhibit remarkable adaptations to prolonged periods of drought and high temperatures and are popular in family beekeeping (11). Malícia honey possesses unique sensory and nutritional characteristics, containing linalool as the main volatile compound, which is characteristic of this honey (12). It also contains organic acids such as acetic, lactic, and tartaric acids; glucose, fructose, and maltose as the primary sugars; and major phenolic compounds, including 3,4-hydroxybenzoic acid, procyanidin B1 and B2, epicatechin, naringenin, kaempferol, quercetin, and myricetin (13, 14). These constituents contribute to the honey’s antioxidant and antimicrobial properties *in vitro*, as well as to its previously reported hypolipidemic, hypoglycaemic, neurobehavioural, and intestinal and liver health-modulating activities *in vivo*. Furthermore, previous studies have shown the bioactive effect of malícia honey in dyslipidaemic and obese rodent models (9, 10). Malícia honey (*Mimosa quadrivalvis* L.) administration (1,000 mg/kg body weight) in dyslipidaemic Wistar rats for 5 weeks decreased total cholesterol and low-density lipoprotein (LDL), improved colon epithelial integrity and intestinal health; preserved liver cell structure and increased hepatic activity of the enzyme superoxide dismutase (SOD), indicating that this honey has antioxidant potential (9). Moreover, malícia honey administration (100 mg/kg body weight) in cafeteria diet-induced obese Wistar rats for 8 weeks reduced the body mass index (BMI) and adiposity index, improved their lipid profile, leptin, insulin, and glucose metabolism, reduced brain inflammation, and showed antidepressive and anxiolytic effects (10, 14). Thus, it can be observed that malícia honey intake under different pathological conditions has demonstrated protective effects on the intestine, liver, and brain (9, 10).

There is still limited evidence regarding the therapeutic applications and health benefits of malícia honey, and no study has been conducted to date to evaluate the effect of malícia honey consumption focusing on its phenolic compounds on vital organs that are deleteriously affected by obesity. Thus, considering the outstanding nutritional characteristics and bioactive properties of malícia honey against metabolic disorders, the present study aimed to evaluate whether the malícia honey (*Mimosa quadrivalvis* L.) administration to obese rats induced by a cafeteria diet reduced oxidative stress parameters in the brain, gut, kidney, and liver through phenolic metabolic action for the first time in the literature.

## 2 Materials and methods

### 2.1 Phenolic profile of malícia honey

Malícia honey (*Mimosa quadrivalvis* L.) was directly acquired from jandaíra (*Melipona subnitida* D.) beekeepers situated in Sitio Novo city, within the state of Rio Grande do Norte. It is a semi-arid region in Northeast Brazil, positioned at coordinates 6° 06′ 14″ S 35° 54′ 39″ W. The malícia honey samples were gathered during the 2019 dry season. These samples were preserved in sterile amber glass containers, dispatched to the laboratory, and stored at temperatures between 6–8°C in the absence of light until subjected to analysis.

Melissopalynological analysis was conducted to ensure the botanical origin following Barth (15) and this honey was characterised regarding its phenolic profile (16, 17) and organic acids and sugars (14) (Supplementary Table S1) by high-performance liquid chromatography (Agilent 1260 Infinity LC system, Santa Clara, United States) coupled to a diode array detector (DAD) (model G1315D). For the identification and quantification of phenolic compounds Zorbax Eclipse Plus RP-C18 column (100 × 4.6 mm, 3.5 μm) and a Zorbax C18 pre-column (12.6 × 4.6 mm, 5 μm) was used. For the identification and quantification of sugars and acids, an Agilent Hi-Plex H column (300 × 7.7 mm) had a particle size of 8.0 μm and the PL Hi-Plex H guard column (5 × 3 mm) (Agilent Technologies, Santa Clara, California, United States) was used.

### 2.2 Ethical considerations, study design, diets, and honey administration

The experiments were conducted at the Laboratory of Experimental Nutrition (LANEX) at the Federal University of Paraiba (UFPB), located in Paraiba, Brazil. Prior approval was obtained from the Ethics Committee on Animal Use of the Federal University of Paraiba (CEUA/UFPB) under protocol number 4757071019 and the experiment was performed in compliance with the Animal Research: Reporting of *In Vivo* Experiments (ARRIVE) guidelines (18).

A total of 40 male Wistar rats, approximately 50 days old (weighing 228 ± 14.69 g) were utilised in this study. The rats were housed in two rats per cage and provided with unrestricted access to water and diets. The housing conditions were maintained at a temperature of 21 ± 1°C, relative humidity ranging from 50 to 55%, and a 12-h light-dark cycle (with lights off at 7 p.m.).

**Figure 1:**
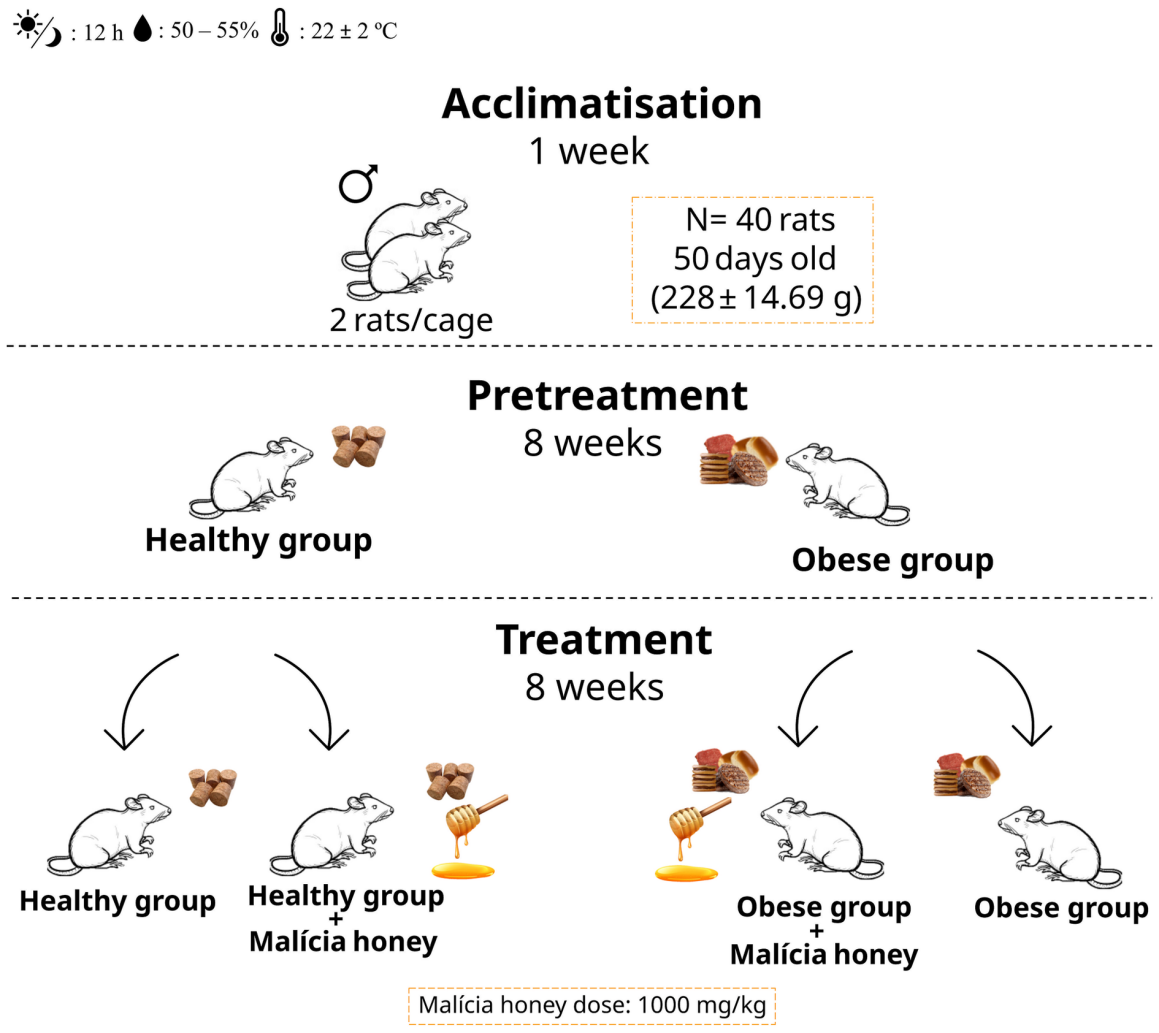
Experimental design. HG, healthy group; HGH, healthy group treated with malícia honey; OG, obese group; OGH, obese group treated with malícia honey.

After an acclimatisation period of 1 week, the Wistar rats were randomly assigned to two main groups: a healthy group (HG, *n* = 20) fed a standard diet and an obese group (OG, *n* = 20) fed a cafeteria diet for the initial 8 weeks. These groups were subsequently further distributed into four subgroups for the final 8 weeks: healthy control (HG, *n* = 10), obese (OG, *n* = 10), healthy group with honey administration (HGH, *n* = 10), and obese group with honey administration (OGH, *n* = 10), according to the experimental design represented in Figure 1.

The OG and OGH groups were induced into obesity through administration of a cafeteria diet, which has been previously validated as an effective method for inducing obesity, mirroring the consumption patterns observed in obese humans (16, 17). This cafeteria diet comprised standard commercial feed (Presence, Paulínea, São Paulo, Brazil) and a daily selection of high-calorie ultra-processed foods (Supplementary Table S2) served in individual glass jars within the cages (19, 20). The cafeteria diet provided 370 kcal/100 g of diet, 55.99 kcal % of carbohydrates, 7.92 kcal % of protein, and 36.10 kcal % of lipids.

Meanwhile, the HG and HGH groups were housed in cages with empty glass jars and received the same standard commercial feed (Presence, Paulínea, São Paulo, Brazil) provided in the cage lid to maintain consistent conditions. The control diet provided 320 kcal/100 g of diet, 76.31 kcal % of carbohydrates, 14.53 kcal % of proteins, and 10.10 kcal % of lipids. These respective diets were administered for 16 weeks.

Commercial feed was offered on the cage lid to all four groups. Only two rats were accommodated in each cage due to the space occupied by the glass jars (20, 21), and also to minimise potential stress and behaviour change caused by loneliness over the 16 weeks of experiment, considering that rats are very social animals that prefer to stay with companions (22).

Malícia honey (*Mimosa quadrivalvis* L.) was administered daily to the HGH and OGH groups at a dose of 1,000 mg/kg body weight (2 mL) via the orogastric route during the final 8 weeks of the experiment (9, 23). The HG and OG control groups were administered with filtered water via the same route and volume (2 mL) as honey to simulate the same condition among all groups. The honey dose (1,000 mg/kg) was determined based on a preliminary study and prior research demonstrating the antioxidant, lipid-lowering, and other therapeutic effects of malícia honey against metabolic alterations induced by obesogenic diets in rodents (9, 10).

Weekly measurements of body weight were performed to monitor weight gain (in grams) and daily assessments were conducted to measure food intake (in kcal) throughout the experiment. Daily diet intake was assessed at the same time (11 a.m.) and was represented by the difference in grams between the diet offered (control or cafeteria) and the residual diet collected daily inside the cage or left on the cage lid.

Total energy intake (kcal) was calculated in kcal based on the labelling and consumption of ultra-processed foods plus the feed for the cafeteria diet or only the feed for the control diet, considering the energy provided by the macronutrients and the Atwater conversion factors (24).

### 2.3 Euthanasia and collection of biological materials

At the end of the experiment, the rats were fasted for 6 h and then euthanised by guillotine decapitation (EB271, Insights, Ribeirão Preto, Brazil) to collect brain, gut, kidney, and liver to perform analyses of oxidative parameters, quantification of phenolic compounds and histology.

The collected organs were washed in saline solution (0.9% NaCl) and then frozen at −80°C for later analysis of oxidative parameters and phenolic compounds. The organs intended for histological analysis were immersed in a 10% buffered formaldehyde solution for 48 h and subsequently processed.

### 2.4 Oxidative parameters in brain, gut, kidney and liver

Lipid peroxidation by quantification of thiobarbituric acid reactive substances (TBARS) was assessed in the brain, gut, kidney, and liver, and expressed in malondialdehyde content (MDA) (25). The total antioxidant capacity (TAC) was measured in the same organs following the method proposed by Brand-Williams et al. (26) with modifications suggested by Zarban et al. (27) using the 2,2-diphenyl-1-picrylhydrazyl (DPPH) free radical scavenging assay. A DPPH control solution was prepared at 0.06 mM by dissolving 0.0012 g of DPPH in 50 mL of ethanol. The absorbance of the DPPH control solution was measured using a spectrophotometer (Femto 700S, São Paulo, Brazil) at a wavelength of 517 nm, with ethanol PA used for calibration. Thus, 50 μL of each sample was mixed with 1 mL of DPPH solution in ethanol (0.06 mM) for the determinations. The mixture was homogenised and left to stand for 30 min in a water bath at 37°C. Then, 0.5 mL of chloroform was added, and the mixture was centrifuged at 7,871 × g for 5 min.

### 2.5 Quantification of phenolic compounds in brain, gut, kidney and liver

The extraction of phenolic compounds was performed in the brain, liver, gut, and kidney following the methodology of Chen et al. (28) adapted by Batista et al. (29).

HPLC (model 1260 Infinity, Agilent Technologies, Santa Clara, CA, United States) equipped with a diode array detector (DAD; model G1315D), column Zorbax Eclipse Plus RP-C18 (100 × 4.6 mm, 3.5 μm) and Zorbax C18 guard column (12.6 × 4.6 mm, 5 μm) (Agilent Technologies, Santa Clara, CA, United States) was used to identify and quantify phenolic compounds in organs. The following analytical conditions were used for this analysis: oven temperature 35°C, solvent flow 0.8 mL/min; gradient used in the 0–5 min separation was 5% solvent B (methanol acidified with 0.5% H_3_PO_4_), 5–14 min (23% B), 14–30 min (50% B), and 30–33 min (80% B). Compounds were detected at 220, 280, 320, 360, and 520 nm, and identified and quantified by comparison with external standards (29).

External standards for phenolic compounds (gallic acid, p-coumaric acid, chlorogenic acid, syringic acid, trans-caftaric acid, caffeic acid, hesperidin, naringenin, procyanidin B1, catechin, epicatechin, and procyanidin B2) were also purchased from Sigma-Aldrich (St. Louis, MO, United States). External standards for procyanidin A2, epigallocatechin gallate, epicatechin gallate, kaempferol 3-glucoside, quercetin 3-rutinoside (rutin), quercetin 3-glucoside, and myricetin were obtained from Extrasynthese (Genay, France). Cis-Resveratrol and trans-resveratrol were purchased from Cayman Chemical Company (Ann Arbor, MI, United States).

### 2.6 Histological analysis in brain, gut, kidney and liver

The brain (hippocampus), gut (colon), left kidney, and liver (left lobe) were fixed in 10% buffered formaldehyde and processed according to routine histological technique. The slides obtained were stained using the Harris Haematoxylin and Eosin (H&E) technique, performing the assembly between slide and coverslip with synthetic resin (Entellan^®^, Merck, Darmstadt, HE, Germany) for analysis in increasing objectives and photographed with increasing 20 x magnifications in a standard optical microscope (Motic BA 200, Santa Monica, United States). The slides were reassessed by the same pathologist to confirm the observations after being randomised by an independent person and the general agreement between the two analyses was considered as an evaluation criterion (30).

### 2.7 Statistical analysis

The data were submitted to the Kolmogorov–Smirnov test to evaluate the normal distribution and Levene’s test to analyse the variance homogeneity. Parametric data from two groups and four groups were, respectively, submitted to the Student’s *t*-test (*p* ≤ 0.05) and analysis of variance (ANOVA), followed by Tukey’s post-test at a significance level of 5% (*p* ≤ 0.05) when there was a difference between groups. The GraphPad Prism 8 free software (GraphPad Software, La Lolla, CA, United States) was used for univariate statistical methods. The MetaboAnalyst v.6.0 free software (Xia Lab, McGill University, Montreal, Canada) was used for creating heatmaps.

## 3 Results

### 3.1 Evaluation of energy intake and the body weight of rats

**Figure 2:**
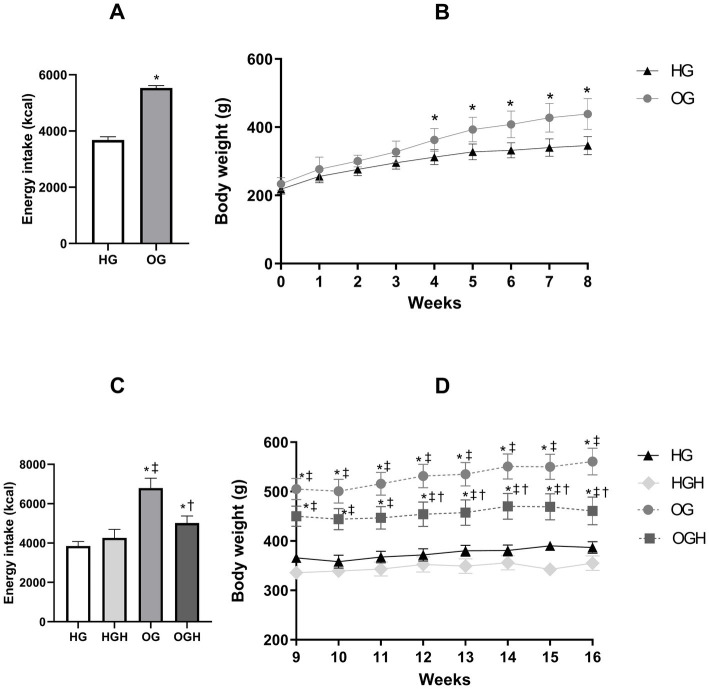
Total energy intake (kcal) and total weight gain (g) of healthy and obese rats treated or not with malícia honey in the pre-treatment period (**A,B**, respectively) and treatment period (**C,D**, respectively). HG, healthy group (*N* = 10); HGH, healthy group treated with malícia honey (*N* = 10); OG, obese group (*N* = 10); OGH, obese group treated with malícia honey (*N* = 10). Results were presented as mean and standard deviation and were evaluated by Student’s *t*-test or one-way ANOVA and Tukey’s post-test, *p* ≤ 0.05. ^*^Difference compared with the HG. ^‡^Difference compared with the HGH. ^†^Difference compared with the OG.

The obese group (OG) showed higher energy intake (Figures 2A,C) in both phases of the experiment (pre-treatment and treatment) compared to the healthy control group (HG) (*p* ≤ 0.05). The body weight of OG increased significantly in the last 5 weeks of pre-treatment period (Figure 2B) and in the 8 weeks of treatment period (Figure 2D), compared with the body weight of HG (*p* ≤ 0.05). Meanwhile, malícia honey administration was able to reduce energy intake (Figure 2C) and body weight of OGH rats (Figure 2D) in last 5 weeks of the treatment phase compared to OG rats (*p* ≤ 0.05).

### 3.2 Effects on total antioxidant capacity in organs of rats

**Figure 3:**
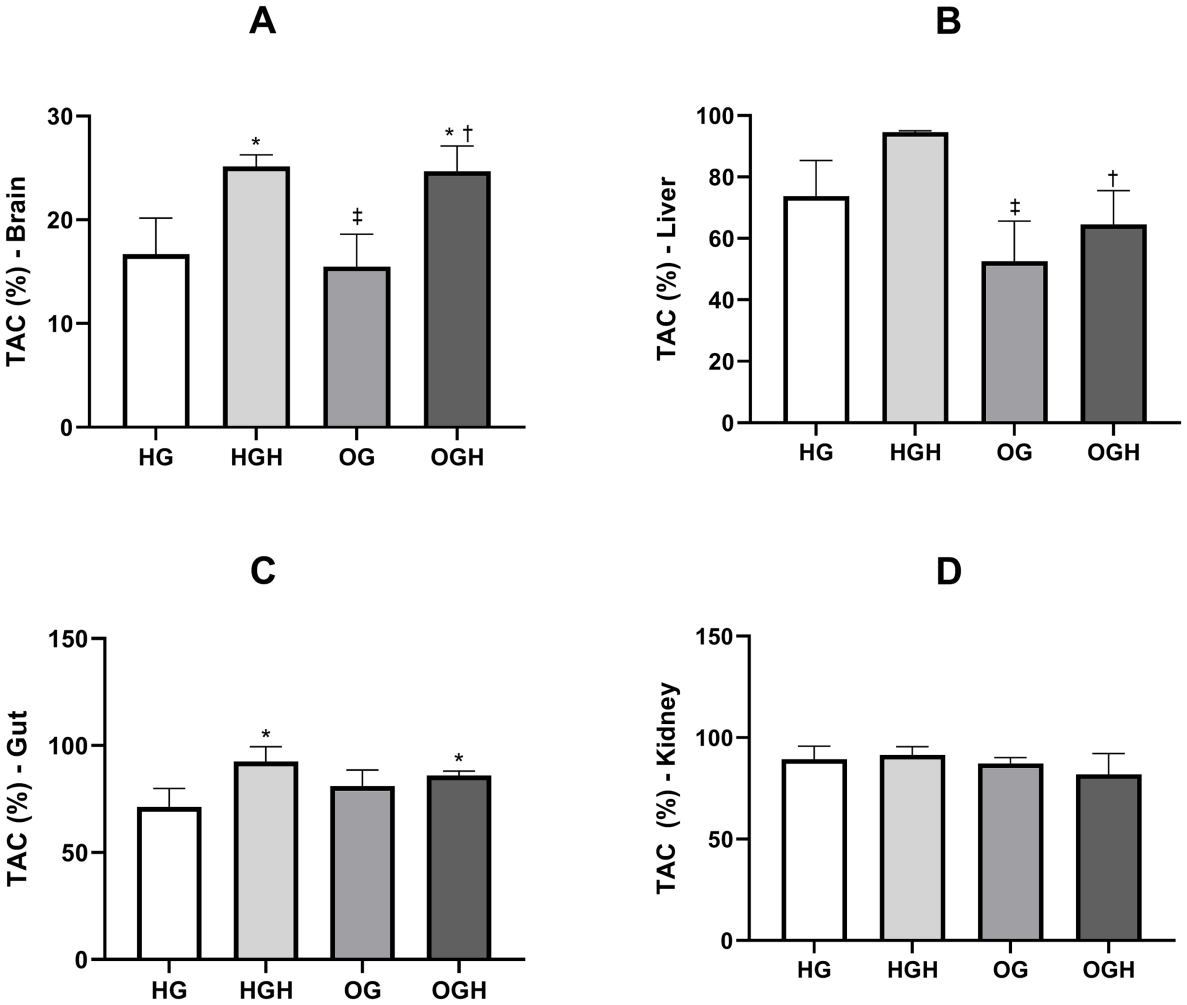
Total antioxidant capacity of brain **(A)**, liver **(B)**, gut **(C)**, and kidney **(D)** of healthy and obese rats treated or not with malícia honey. HG, healthy group (*N* = 10); HGH, healthy group treated with malícia honey (*N* = 10); OG, obese group (*N* = 10); OGH, obese group treated with malícia honey (*N* = 10). Results are presented as mean and standard deviation and were evaluated by one-way ANOVA, *p* ≤ 0.05, Tukey’s post-test. ^*^Difference compared to the HG. ^‡^Difference compared to the HGH. ^†^Difference compared to the OG.

The total antioxidant capacity (TAC) in the brain, gut, kidney, and liver is shown in Figure 3. The HGH (25.15 ± 1.11%) and OGH (24.70 ± 2.42%) showed higher total antioxidant capacity in the brain (Figure 3A) compared to the HG (16.70 ± 3.47%) and OG (15.48 ± 3.14%), respectively (*p* ≤ 0.05). Malícia honey treatment increased TAC in the liver (Figure 3B) in the OGH (64.50 ± 11.07%) compared to OG (52.53 ± 13.07%) (*p* ≤ 0.05). In turn, the groups treated with malícia honey regardless of the diet consumed showed higher TAC (HGH = 92.60 ± 6.89%; OGH = 85.95 ± 2.17%) in the gut (Figure 3C) compared to the HG control (71.33 ± 8.64%) (*p* ≤ 0.05). However, there was no significant difference in TAC among the groups in the kidney (Figure 3D) (*p* > 0.05).

### 3.3 Effects on lipid peroxidation in organs of rats

**Figure 4:**
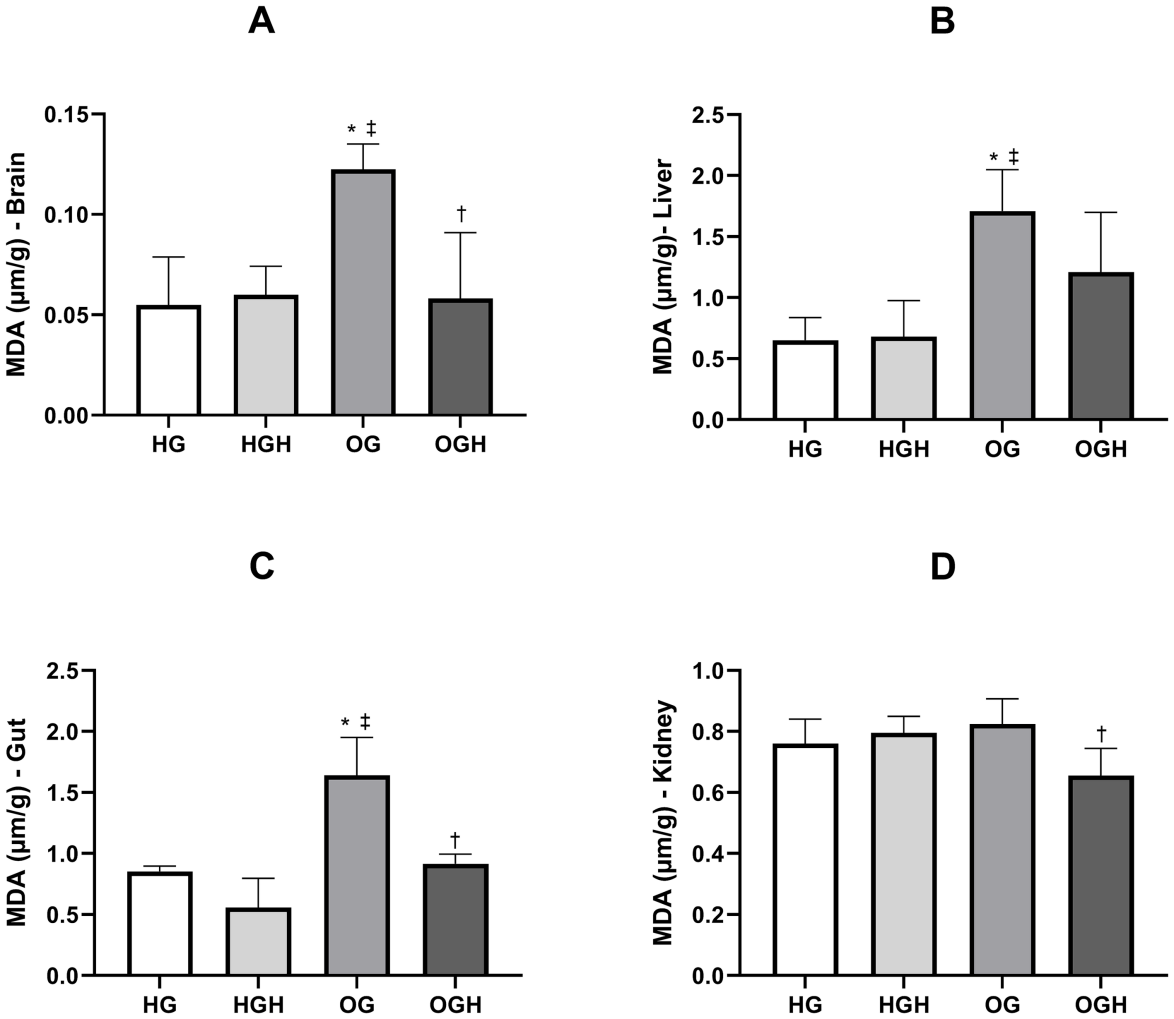
Malondialdehyde (MDA) of brain **(A)**, liver **(B)**, gut **(C)**, kidney **(D)** of healthy and obese rats treated or not with malícia honey. HG, healthy group (*N* = 10); HGH, healthy group treated with malícia honey (*N* = 10); OG, obese group (*N* = 10); OGH, obese group treated with malícia honey (*N* = 10). Results are presented as mean and standard deviation and was evaluated by one-way ANOVA, *p* ≤ 0.05, Tukey’s post-test. ^*^Difference compared to the HG. ^‡^Difference compared to the HGH. ^†^Difference compared to the OG.

Lipid peroxidation was quantified by malondialdehyde (MDA) formation in the brain, liver, gut, and kidney, as shown in Figure 4. The OG showed greater lipid peroxidation in the brain (0.12 ± 0.01 μm/g) (Figure 4A), liver (1.71 ± 0.34 μm/g) (Figure 4B) and gut (1.64 ± 0.31 μm/g) (Figure 4C) compared to the HG (0.06 ± 0.02 μm/g of brain; 0.65 ± 0.19 μm/g of liver; 0.85 ± 0.04 μm/g of gut) (*p* ≤ 0.05). Malícia honey treatment was able to reduce lipid peroxidation in the brain (0.06 ± 0.03 μm/g) (Figure 4A), gut (0.92 ± 0.08 μm/g) (Figure 4B) and kidney (0.66 ± 0.09 μm/g) (Figure 4D) of the OGH when compared to the same organs in the OG (0.12 ± 0.01 μm/g of brain; 1.64 ± 0.31 μm/g of gut; 0.83 ± 0.08 μm/g of kidney) (*p* ≤ 0.05).

### 3.4 Phenolic compounds quantified in brain, liver, gut, and kidney tissues

**Figure 5:**
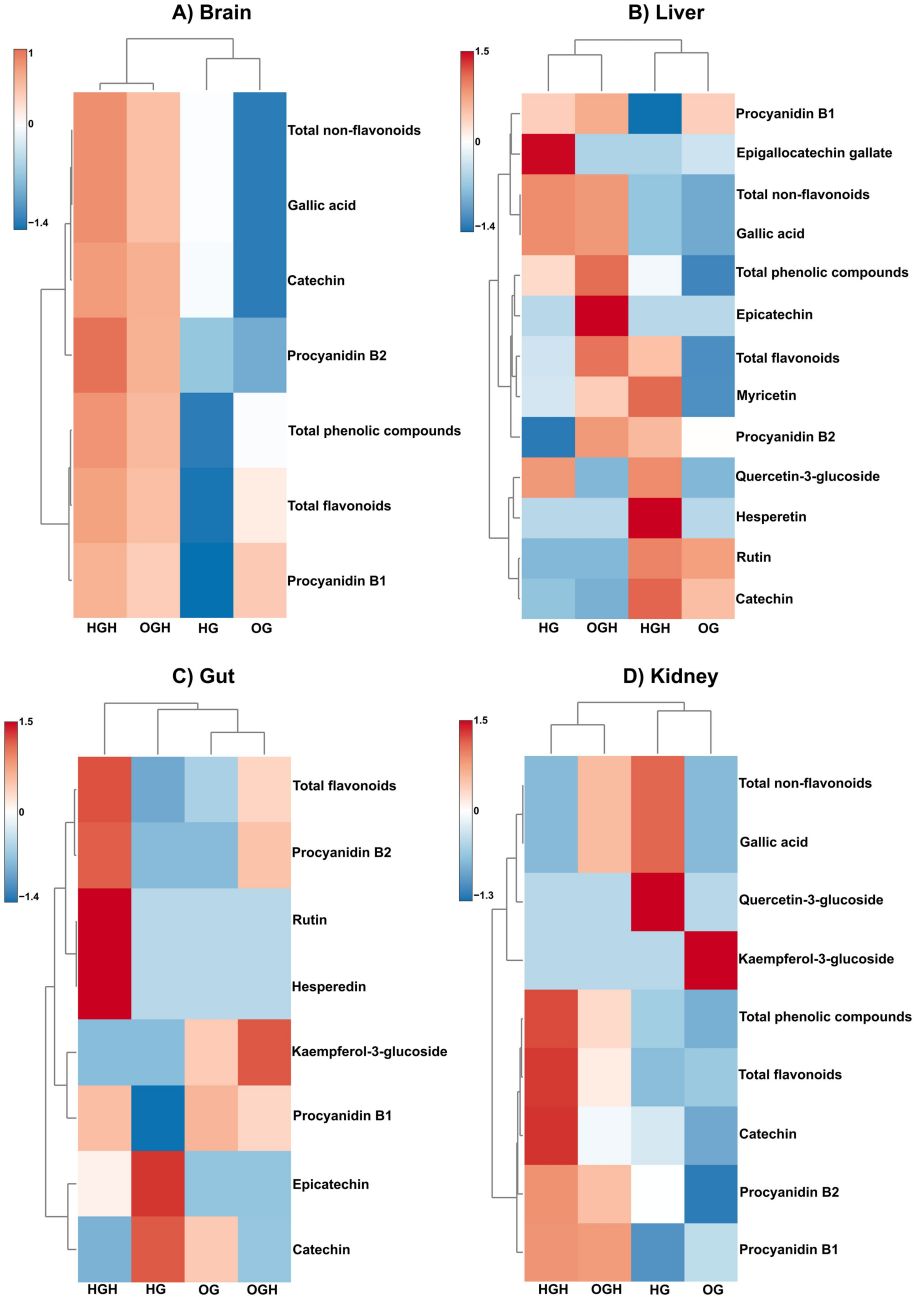
Hierarchical clustering via heatmap using Euclidean as a distance measure and Ward as clustering algorithm of phenolic compounds in brain **(A)**, liver **(B)**, gut **(C)** and kidney **(D)** of rats. HG, healthy group (*N* = 10); HGH, healthy group treated with malícia honey (*N* = 10); OG, obese group (*N* = 10); OGH, obese group treated with malícia honey (*N* = 10).

The phenolic compound deposition was higher in the brains of the HGH rats compared to the HG group (*p* ≤ 0.05) (Figure 5A and Supplementary Table S3), particularly in terms of catechin, procyanidin B1 and B2, total flavonoids, gallic acid, and total phenolic compounds. At the same time, the phenolic compound deposition was higher in the brains of the OGH rats compared to the OG group (*p* ≤ 0.05) (Figure 5A and Supplementary Table S3), particularly in terms of catechin, procyanidin B2, total flavonoids, gallic acid, and total phenolic compounds. The HGH showed a higher phenolic compound deposition (procyanidin B1, total flavonoids, gallic acid, and total phenolic compounds) than the OGH (*p* ≤ 0.05) (Figure 5A and Supplementary Table S3).

The phenolic compound deposition was higher in the livers of the HGH rats compared to the HG group (*p* ≤ 0.05) (Figure 5B and Supplementary Table S4), particularly in terms of catechin, procyanidin B2, hesperidin, myricetin, and total flavonoids. At the same time, the phenolic compound deposition was higher in the livers of the OGH rats compared to the OG group (*p* ≤ 0.05) (Figure 5B and Supplementary Table S4), particularly in terms of epicatechin, procyanidin B1 and B2, myricetin, total flavonoids, gallic acid, and total phenolic compounds. The HGH showed a higher phenolic compound deposition of catechin than OGH, while OGH showed a higher deposition of procyanidin B1 and B2, total flavonoids, and total phenolic compounds than the HGH (*p* ≤ 0.05) (Figure 5B and Supplementary Table S4).

Rats that received malícia honey (HGH and OGH) showed higher flavonoid content in gut tissue compared to the other rat groups (*p* ≤ 0.05) (Figure 5C and Supplementary Table S5). Notably, the HGH rats exhibited higher procyanidin B1 and B2, hesperidin, rutin, and total flavonoid levels in the gut than the HG (*p* ≤ 0.05). At the same time, the OGH showed higher procyanidin B2, kaempferol 3-glulcoside and total flavonoid levels than the OG (*p* ≤ 0.05).

Flavonoid and phenolic compound deposition were higher in the kidneys of the HGH and OGH rats (*p* ≤ 0.05) (Figure 5D and Supplementary Table S6), particularly in terms of catechin, procyanidin B1, total flavonoids and total phenolic compounds for the HGH compared to the HG. At the same time, the phenolic compound deposition was higher in the kidneys of the OGH rats compared to the OG (*p* ≤ 0.05), particularly catechin, procyanidin B1 and B2, total flavonoids, gallic acid, and total phenolic compounds.

### 3.5 Evaluation of brain, hepatic, intestinal and renal histology

**Figure 6:**
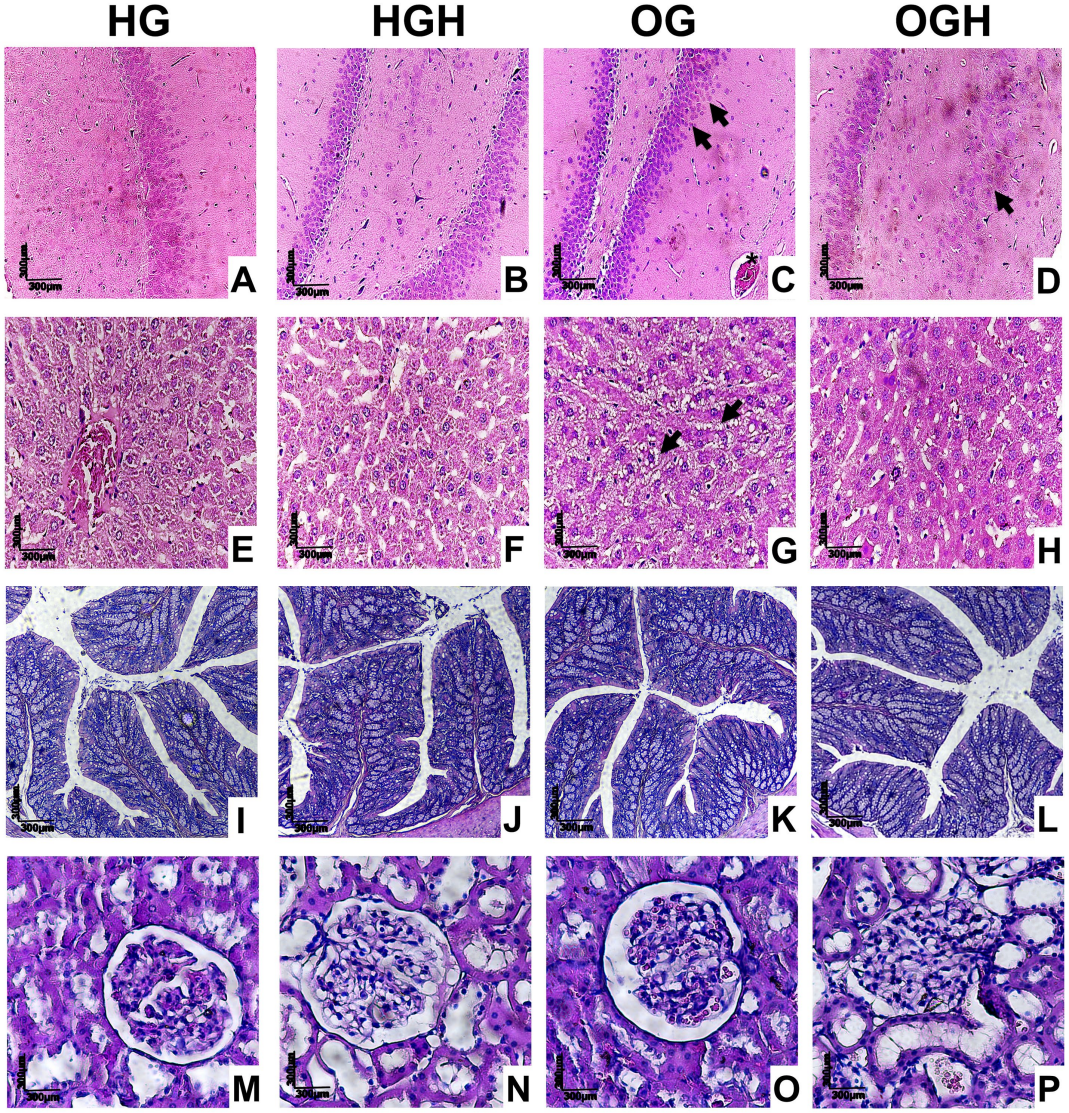
Representative histological sections of hippocampus **(A–D)**, liver **(E–H)**, colon **(I–L)**, and kidney **(M–P)** of healthy and obese rats treated or not with malícia honey. Black arrowhead = pyknotic neurons (brain) or fat accumulation (liver); asterisk = hyperaemia. HG, healthy group (*N* = 10); HGH, healthy group treated with malícia honey (*N* = 10); OG, obese group (*N* = 10); OGH, obese group treated with malícia honey (*N* = 10).

There was neuron maintenance in all regions in the hippocampus (Figures 6A,B) in the HG and HGH. Nevertheless, several pyknotic neurons were observed in the OG group (black arrowhead), in addition to hyperemia (asterisk) (Figure 6C), while few pyknotic neurons were noticed in the OGH rats (Figure 6D). Hepatocyte and hepatic cord maintenance was observed in the liver (Figures 6E–H) in rats from the HG (Figure 6E), HGH (Figure 6F), and OGH (Figure 6F); however, hepatic steatosis was observed in rats from the OG (Figure 6G) (black arrowhead). Preserved parenchyma was observed in the intestinal colon with intestinal villi and crypts maintained without cellular changes in all experimental conditions (Figures 6I–L). Renal glomeruli and glomerular tufts were observed in the analysis of the renal parenchyma, without changes in all experimental conditions (Figures 6M–P).

## 4 Discussion

The present study demonstrated that malícia honey administration reduced caloric intake and body weight in obese rats and improved morphological parameters and parameters related to oxidative stress in the brain, gut, liver, and kidney of healthy and obese rats by depositing phenolic compounds in the tissues. Although honeys are sources of sugar, the lower fructose/glucose ratio of malícia honey compared to other monofloral honeys and the phenolic compounds contained in this honey may contribute to the lower caloric intake observed in obese OGH versus OG rats (10, 14). Consuming a natural source of antioxidant phenolic compounds is related to a decrease in oxidative stress products (e.g., MDA, 8-isoprostane and F2-isoprostane) through hydrogen atom transfer, electron transfer and/or proton loss for free radicals (6, 7). In this scenario, malícia honey, which is rich in phenolic compounds (14), is a very important food which can improve the daily intake of these compounds (8).

Regarding the brain results, malícia honey improved TAC and reduced lipid peroxidation product (MDA) in healthy and obese groups. The phenolic profile in the brains of the HGH and OGH rats showed an increase in catechin, procyanidins B1 and B2, total flavonoids, gallic acid and hydroxybenzoic acid, as well as total phenolic compounds. Furthermore, the OGH rats had no hyperaemia and few neurons with nuclear pyknosis (an irreversible process of apoptotic cell death) (31) compared with the OG rats. Importantly, the hyperaemia observed in the hippocampus of the OG consists of an increase in blood flow above the basal level which may have occurred in response to obesity-induced brain ischemia-reperfusion (32, 33). This reactive hyperaemia causes brain damage through higher free radical production and neutrophil influx which has a special role in chronic inflammation (32, 34).

Several studies have demonstrated the negative impact of diet-induced obesity on oxidative stress and inflammation in the brain (35, 36). Nevertheless, the antioxidant capacity and anti-inflammatory potential of malícia honey and other stingless bee honey protected the brains of obese rats from tissue damage. Malícia honey intake (1,000 mg/kg body weight) reduced neuroinflammation through lower brain nuclear factor kappa B (NF-κB) and serum C-reactive protein in obese rats, and also showed antidepressive and anti-anxiolytic effects associated with antioxidant and anti-inflammatory potential of their phenolic compounds (10, 14). Administration of honey from *Heterotrigona itama* bees (1 g/kg body weight) in metabolic syndrome-induced rats decreased brain pro-inflammatory tumour necrosis factor-alpha (TNF-α) and increased brain-derived neurotrophic factor essential for memory and learning (37).

Isolated flavonoids and hydroxybenzoic acids, especially gallic acid, also confer brain health (38). Ramis et al. (39) observed that flavanol catechin improved cognitive function by increasing the accumulation of 5-hydroxytryptophan and dihydroxyphenylalanine and regulated the neuroinflammation by reducing sirtuin 1 protein expression in the hippocampus of rats aged 18 months. Procyanidins, including B1 and B2 forms, protect rat neurons from oxidative stress by inhibiting the ROS and MDA formation; increasing glutathione peroxidase, SOD, and catalase activities; and up-regulating the nuclear factor-erythroid 2-related factor 2 (Nrf2)/antioxidant response element pathway (40). Furthermore, gallic acid has been reported to contribute to neuroprotection and memory improvement in rats with metabolic syndrome; the gallic acid could protect the hippocampus dendritic morphology, with a reduction of ROS production and proinflammatory cytokines in this brain area (41). These could explain the improvement of cell lipid peroxidation and hippocampus integrity in the OGH group, which had high catechin, procyanidin B2, and gallic acid concentrations in the brain.

The brain health benefits caused by phenolic compounds (e.g., catechins, procyanidins, and gallic acid) are due to their ability to accumulate in cerebrospinal fluid and brain tissue (42–46). However, the mechanisms by which phenolic compounds reach the brain are not yet fully understood. Some authors (42–44) suggest that chemical modifications, such as methylation, enable phenolic compounds and their metabolites to cross the blood-brain barrier. Phenolic compound transport may occur through passive diffusion or transporter-mediated processes, such as ATP-binding cassette protein-type efflux transporters (42–44).

The obese group had an increase in lipid oxidative damage and hepatic steatosis in the liver. Malícia honey treatment improved the total antioxidant capacity and reversed hepatic steatosis in obese rats, even without significantly reducing liver MDA values. The livers of the rats that received malícia honey presented high epicatechin (OGH), procyanidin B1 (OGH), procyanidin B2 (HGH and OGH), hesperedin (HGH) and myricetin (HGH) concentrations. Isolated hesperidin and hesperidin aglycone showed hepatic protection against non-alcoholic fatty liver disease (NAFLD) by decreasing the ROS and MDA values; increasing the antioxidant activity of SOD, glutathione peroxidase, glutamate-cysteine ligase catalytic subunit, glutathione reductase and heme oxygenase-1; and controlling liver inflammation through suppression of NF-κB activation (47).

Myricetin reduced lipid accumulation and lipid hydroperoxide, lipid peroxide and protein carbonyl in the livers of C57BL/6J mice with high-fat diet-induced obesity (48). Epicatechin treatment in the liver of highly fat-fed mice reduced the expression of NADPH oxidase isoforms, such as NOX3 and NOX4, responsible for cell production of free radical superoxide and hydrogen peroxide. Epicatechin even promotes lower lipid deposition and NOX3/NOX4 expression in a human hepatocellular cancer cell line incubated with palmitate (49).

Additionally, procyanidin B1 improved liver lipid metabolism, reduced cholesterol, LDL, and triglycerides, increased HDL, and decreased inflammation through activating proliferator-activated receptor alpha in the liver of diabetic rats (50). *In vitro* (human hepatic stellate cell) and *in vivo* (mice with CCl4-induced liver fibrosis) analysis demonstrated that procyanidin B2 has protective effects against liver fibrosis by inhibiting the Hedgehog signalling pathway responsible for tissue development and remodelling (51). This effect results from lower expression of key molecules of this pathway, such as smoothened, glioma-associated oncogene homolog 1, hypoxia-inducible factor 1-alpha, vascular endothelial growth factor A, α-smooth muscle actin, constans-like 1 and growth factor-beta 1 (51).

Cheng et al. (52) observed that the consumption of stingless bee (*Heterotrigona itama*) honey (0.25 g/kg or 0.5 g/kg) or its phenolic-rich extract (12.5 mg/kg or 25 mg/kg) by high-fat diet fed streptozotocin-nicotinamide-induced diabetic rats for 50 days conferred a higher total antioxidant status, lower oxygen radical absorbance capacity, lower SOD activity resulting from the reduction of ROS generation caused by phenolic compounds, as well as lower MDA and 8-iso-PGF2α (product of arachidonic acid peroxidation) content in their livers. The authors also detected upregulation of antioxidant genes in the liver in response to both treatments, such as *Nrf2*, *superoxide dismutase 1* and *heme oxygenase 1*: involved in kelch-like ECH-associated protein 1: *Nrf2* pathway, mainly a protective response to oxidative stress (52).

The gut and kidney of the OGH had lower MDA concentrations than the OG, without histological changes in these tissues, demonstrating a protective effect of malícia honey against obesity tissue damage, especially due to their higher flavonoid concentrations in contrast to the OG; for example, catechin (kidney), procyanidins B1 (kidney) and B2 (gut and kidney), and kaempferol-3-glucoside (gut).

Catechin significantly reduces the ROS levels in kidney tissue in a mouse sepsis model (one of the main factors contributing to kidney damage during sepsis), attenuates macrophage polarization towards the M1 phenotype and decreases proinflammatory cytokine (TNF-α, interleukins IL-1β and IL-6) in mice sepsis models and macrophage cell cultures (44). In addition, catechin administration showed less histological damage to the kidneys compared with non-treated septic mice, with a reduction in cell apoptosis and preservation of kidney function, as indicated by lower serum creatinine and urea levels (53).

Procyanidins B1 and B2 neutralised free radicals to protect human kidney cells against hydrogen peroxide or hypoxia-induced cell death (54). *In vitro* analysis demonstrated that procyanidin B2 has a protective effect against hyperglycaemia-induced mesangial cell dysfunction in the kidney, with a decrease of oxidative stress and cellular inflammation by inactivating redoxosome signalling pathway (as TGF-β1/SMAD and IL-1β/TNF-α/NF-κB); this mechanism was dependent on caveolin-1 (CAV-1) suppression in these cells (55). Peanut skin procyanidins, including B1 and B2 forms, offered to type 2 diabetic mice preserved villus length and crypt depth, improved the gut barrier integrity by enhancing colon tight junction protein expression including zonula occludens-1, claudin-1 and occludin (56). Kaempferol-3-glucoside is a substrate for lactase phlorizin hydrolase activity, an intestinal membrane-bound enzyme that hydrolyses lactose and maintains gut health (57, 58).

In our previous study, malícia honey (1,000 mg/kg for 35 days) reversed epithelial destruction and goblet cell destruction in the colons of rats with diet-induced dyslipidaemia (9). Furthermore, stingless bee honey (2 g/kg for 12 days) demonstrates potential for renal protection in streptozotocin-induced diabetic rats, with a decrease in systemic oxidative stress, serum urea and creatinine, and decreased hydropic changes caused by diabetes in the kidney architecture (59).

As limitations of our study, we believe it would be important to include an analysis of phenolic compounds and their metabolites in plasma to also verify the systemic action of these compounds, as well as an analysis of the influence of acute and chronic ingestion of honey on the profile of these compounds to determine potential metabolic pathways in specific axes of our organism. Further studies, including molecular pathways, could be conducted to evaluate the effect of malícia honey consumption on metabolomics and the regulation of protein expression (through immunoblot or enzyme-linked immunosorbent assay—ELISA), or on gene regulation through real-time polymerase chain reaction (qPCR) related to oxidative stress. For future translation studies, the dose of malícia honey administered to rats (1,000 mg/kg) can be calculated based on an equation that considers the body surface of rodents and humans, obtaining 162.16 mg/kg as the equivalent human dose (10, 60).

## 5 Conclusion

The present study demonstrated that administering malícia honey produced by stingless bees reduced caloric intake and weight gain in obese rats fed a cafeteria diet. Furthermore, malícia honey consumption significantly minimised *in situ* oxidative stress in vital organs and increased the deposition of phenolic compounds, such as procyanidins, flavonoids, and gallic acid, in the brain, intestine, liver, and kidneys of these rats. These findings highlight the therapeutic potential of malícia honey as a natural intervention for managing oxidative stress in obesity-related conditions. Future research should aim to unravel the precise molecular pathways underlying the observed effects, evaluate long-term outcomes, and investigate the applicability of these results in human populations. Furthermore, exploring the use of malícia honey in other oxidative stress-related diseases may expand its potential as a functional food in clinical and nutritional strategies.

## Data Availability

The raw data supporting the conclusions of this article will be made available by the authors, without undue reservation.
