# Supplementary material for: Malícia honey (Mimosa quadrivalvis L.) produced by the jandaíra bee (Melipona subnitida D.) shows antioxidant activity via phenolic compound action in obese rats
Source: Front Nutr. 2025 Feb 6;12:1524642. doi: 10.3389/fnut.2025.1524642 (PMC11839446; doi:10.3389/fnut.2025.1524642)
Supplement: Supplementary file 1 [file Data_Sheet_1.docx]

Supplementary Material

**Supplementary Table S1.** Phenolic compounds, organic acids and sugars quantified in the malícia (*Mimosa quadrivalvis* L.) honey produced by the jandaíra bee (*Melipona subnitida* D.) (1)

| **Phenolic compounds (mg/100g)** | **Mean ± standard deviation** | |
| --- | --- | --- |
| *Flavanols and Procyanidins* | | |
| Epicatechin | | 14.34 ± 0.99 |
| Epicatechin gallate | | 9.43 ± 0.17 |
| Epigallocatechin gallate | | 2.41 ± 0.23 |
| Procyanidin A2 | | 4.50 ± 0.49 |
| Procyanidin B1 | | 19.24 ± 0.01 |
| Procyanidin B2 | | 15.90 ± 0.01 |
| *Flavanones* | |  |
| Hesperidin | | 7.41 ± 0.84 |
| Naringenin | | 11.99 ± 0.19 |
| *Flavonols* | |  |
| Kaempferol-3-glucoside | | 2.54 ± 0.01 |
| Myricetin | | 5.90 ± 0.01 |
| Quercetin 3-glucoside | | 2.51 ± 0.03 |
| *Hydroxybenzoic acids* | |  |
| Gallic acid | | 6.04 ± 1.85 |
| *Hydroxycinnamic acids* | |  |
| Caffeic acid | | 9.20 ± 0.83 |
| *Total non-flavonoids* | | 17.91 ± 2.38 |
| *Total phenolic compounds* | | 114.08 ± 4.34 |
| **Organic acids (g/100 g)** | | **Mean ± standard deviation** |
| Acetic acid | | 46.00 ± 0.01 |
| Citric acid | | 3.30 ± 0.01 |
| Formic acid | | 1.00 ± 0.01 |
| Lactic acid | | 192.66 ± 0.04 |
| Malic acid | | 3.10 ± 0.01 |
| Propionic acid | | 1.00 ± 0.01 |
| Tartaric acid | | 0.30 ± 0.01 |
| **Sugars** | | **Mean ± standard deviation** |
| Glucose | | 74.60 ± 0.04 |
| Fructose | | 74.30 ± 0.03 |
| Maltose | | 21.90 ± 0.21 |

**Supplementary Table S2.** Daily menu of the cafeteria diet offered to healthy and obese rats treated or not with malícia honey.

| **Days** | **Menu** |
| --- | --- |
| Monday | Commercial chow, Sandwich biscuit, Marshmallow, Milk sweet and Processed meat. |
| Tuesday | Commercial chow, Wafer, White chocolate, Cheese and Hot dog sausage. |
| Wednesday | Commercial chow, Starch cookie, Sugary corn flakes, Gum drops and Hamburger. |
| Thursday | Commercial chow, White bread, Chocolate, Coconut condensed milk dessert and Pepperoni sausage. |
| Friday | Commercial chow, Chocolate cake, Chocolate corn flakes, Sweet guava and mortadella (bologna). |
| Saturday | Commercial chow, Loaf bread, Stuffed cookie, Chocolate candies, Corn chips, and Ham. |
| Sunday | Commercial chow, Wafer with creamy chocolate filling and Corn chips |

**Supplementary Table S3**. Phenolic compounds concentration in the brain of rats.

| **Phenolic compounds (mg/100g)** | **HG** | **HGH** | **OG** | **OGH** |
| --- | --- | --- | --- | --- |
| *Flavanol and Procyanidins* |  |  |  |  |
| Catechin | 79.16±2.01 | 85.22±2.18* | 69.51±0.00*‡ | 84.17±0.56*† |
| Procyanidin B1 | ND | 192.95±2.10 | 178.90±0.37‡ | 177.10±1.39‡ |
| Procyanidin B2 | 57.71±5.39 | 85.78±0.00* | 53.82±1.10‡ | 79.19±1.06*† |
| Total flavonoids | 136.87±7.40 | 363.94±4.28* | 302.23±0.73*‡ | 340.46±0.24*‡† |
| *Hydroxybenzoic acids* |  |  |  |  |
| Gallic acid | 44.41±0.67 | 57.69±1.62* | 24.30±2.10*‡ | 52.51±1.39*‡† |
| *Total non-flavonoids* | 44.41±0.67 | 57.69±1.62* | 24.30±2.10*‡ | 52.51±1.39*‡† |
| *Total phenolic compounds* | 181.27±6.73 | 421.63±5.90* | 326.53±1.37* ‡ | 392.96±1.63*‡† |

HG= healthy group (N=10); HGH= healthy group treated with malícia honey (N=10); OG= obese group (N=10); OGH= obese group treated with malícia honey (N=10). Results are presented as mean and standard deviation and was evaluated by one-way ANOVA, p≤0.05, Tukey post-test. *difference compared to the HG; ‡ difference compared to the HGH; † difference compared to the OG.

**Supplementary Table S4**. Phenolic compounds concentration in the liver of rats.

| **Phenolic compounds (mg/100g)** | **HG** | **HGH** | **OG** | **OGH** |
| --- | --- | --- | --- | --- |
| *Flavanols and Procyanidins* |  |  |  |  |
| Catechin | 61.66±2.39 | 76.93±2.37* | 72.11±7.20* | 60.20±0.23‡† |
| Epicatechin | ND | ND | ND | 197.27±2.78 |
| Epigallocatechin gallate | 180.64±8.75 | ND | 22.56±0.08* | ND |
| Procyanidin B1 | 170.91±6.19 | 19.89±0.63* | 170.84±6.54‡ | 193.05±3.55* ‡† |
| Procyanidin B2 | 220.24±0.94 | 306.34±1.59* | 281.72±2.91*‡ | 316.52±9.12*‡† |
| *Flavanone* |  |  |  |  |
| Hesperetin | ND | 179.77±4.69 | ND | ND |
| *Flavonols* |  |  |  |  |
| Myricetin | 49.44±9.79 | 113.63±3.89* | ND | 82.56±2.18*† |
| Quercetin-3-glucoside | 23.70±0.30 | 24.58±1.18 | ND | ND |
| Rutin |  | 73.62±1.69 | 68.03±2.90‡ |  |
| *Total flavonoids* | 706.60±28.36 | 794.76±2.02* | 615.25±0.74*‡ | 849.60±7.48 *‡† |
| *Hydroxybenzoic acids* |  |  |  |  |
| Gallic acid | 187.68±0.38 | 30.01±1.57* | ND | 180.08±0.82*† |
| *Total non-flavonoids* | 187.68±0.38 | 30.01±1.57* | - | 180.08±0.82*† |
| *Total phenolic compounds* | 894.28±28.74 | 824.78±3.59* | 615.25±0.74*‡ | 1029.69±8.30*‡† |

HG= healthy group (N=10); HGH= healthy group treated with malícia honey (N=10); OG= obese group (N=10); OGH= obese group treated with malícia honey (N=10). Results are presented as mean and standard deviation and was evaluated by one-way ANOVA, p≤0.05, Tukey post-test. *difference compared to the HG; ‡ difference compared to the HGH; † difference compared to the OG. ND = non-detected.

**Supplementary Table S5**. Phenolic compounds concentration in the gut of rats.

| **Phenolic compounds (mg/100g)** | **HG** | **HGH** | **OG** | **OGH** |
| --- | --- | --- | --- | --- |
| *Flavanols and Procyanidins* |  |  |  |  |
| Catechin | 131.76±3.95 | 80.21±0.04* | 113.40±4.47*‡ | 84.57±3.36*‡† |
| Epicatechin | 55.28±4.97 | 25.41±1.10* | ND | ND |
| Procyanidin B1 | 22.68±2.05 | 180.00±7.32* | 185.77±6.47* | 164.48±1.63*‡† |
| Procyanidin B2 | ND | 437.64±0.57 | ND | 312.12±7.16‡ |
| *Flavanone* |  |  |  |  |
| Hesperidin | ND | 29.66±0.81 | ND | ND |
| *Flavonols* |  |  |  |  |
| Kaempferol-3-glucoside | ND | ND | 30.02±4.31 | 45.36±2.03† |
| Rutin | ND | 116.82±1.03 | ND | ND |
| *Total flavonoids* | 209.73±10.97 | 869.74±7.67* | 329.19±6.31*‡ | 606.53±3.40*‡† |

HG= healthy group (N=10); HGH= healthy group treated with malícia honey (N=10); OG= obese group (N=10); OGH= obese group treated with malícia honey (N=10). Results are presented as mean and standard deviation and was evaluated by one-way ANOVA, p≤0.05, Tukey post-test. *difference compared to the HG; ‡ difference compared to the HGH; † difference compared to the OG. ND = non-detected.

| **Phenolic compounds (mg/100g)** | **HG** | **HGH** | **OG** | **OGH** |
| --- | --- | --- | --- | --- |
| *Flavanol and Procyanidins* |  |  |  |  |
| Catechin | 95.37±1.67 | 193.94±6.55* | 53.35±5.96*‡ | 107.26±0.00*‡† |
| Procyanidin B1 | ND | 183.79±0.94 | 75.07±9.81‡ | 179.92±4.81† |
| Procyanidin B2 | 31.32±6.19 | 47.80±5.28 | ND | 41.49±1.24 |
| *Flavonols* |  |  |  |  |
| Kaempferol-3-glucoside | ND | ND | 38.16±1.10 | ND |
| Quercetin-3-glucoside | 25.24±0.51 | ND | ND | ND |
| *Total flavonoids* | 151.93±8.36 | 425.53±10.89* | 166.59±16.87‡ | 275.03±59.68 *‡† |
| *Hydroxybenzoic acids* |  |  |  |  |
| Gallic acid | 48.44±1.14 | ND | ND | 36.55±2.58* |
| *Total non-flavonoids* | 48.44±1.14 | - | - | 36.55±2.58* |
| *Total phenolic compounds* | 200.37±9.50 | 425.53±10.89 * | 166.59±16.87‡ | 311.58±62.26 *‡† |

**Supplementary Table S6.** Phenolic compounds concentration in the kidney of rats.

HG= healthy group (N=10); HGH= healthy group treated with malícia honey (N=10); OG= obese group (N=10); OGH= obese group treated with malícia honey (N=10). Results are presented as mean and standard deviation and was evaluated by one-way ANOVA, p≤0.05, Tukey post-test. *difference compared to the HG; ‡ difference compared to the HGH; † difference compared to the OG. ND = non-detected.

**Reference**

1. Bezerra MLR, Gouveia-Nhanca M, da Silva Andrade ADA, Pinheiro RO, Alves AF, de Paiva Sousa MC, et al. Malicia honey (*Mimosa quadrivalvis* L.) produced by the jandaíra bee (*Melipona subnitida* D.) improves depressive-like behaviour, somatic, biochemical and inflammatory parameters of obese rats. Food Res Int. (2023) 164:112391. doi:10.1016/j.foodres.2022.112391
